# GENETTA: a network-based tool for the analysis of complex genetic designs

**DOI:** 10.1021/acssynbio.3c00333

**Published:** 2023-11-14

**Authors:** Matthew Crowther, Anil Wipat, Ángel Goñi-Moreno

**Affiliations:** † School of Computing, Newcastle University, Newcastle Upon Tyne, NE4 5TG, United Kingdom; ‡ Centro de Biotecnología y Genómica de Plantas, Universidad Politécnica de Madrid (UPM)- Instituto Nacional de Investigación y Tecnología Agraria y Alimentaria (INIA/CSIC), Madrid, Spain; ¶ School of Computing, Newcastle University, Newcastle Upon Tyne, United Kingdom

**Keywords:** Genetic design, Networks, Data analysis, Knowledge graph, SBOL, Computer-aided design

## Abstract

GENETTA is a software tool that transforms synthetic biology designs into networks using graph theory for analysis and manipulation. By representing complex data as interconnected points, GENETTA allows dynamic customization of visualizations, including interaction networks and parts hierarchies. It can also merge design data from multiple databases, providing a unified perspective. The generated interactive network can be edited by adding nodes and edges, simplifying changes to existing design files. This article presents GENETTA and its features through specific use cases, showcasing its practical applications.

## Introduction and Motivation

Synthetic biology brings engineering and computational concepts and abstractions to molecular biology.^1^ Among these, design principles are fundamental to the synthetic biology lifecycle.^2^ While the complexity of designs increases, the handling of the resulting data (of various types) poses a significant challenge. Design data is not only genetic sequences but information about the function and structure of biological circuits; and this information needs to be captured. The correct handling of design data can add value to the standardisation^3^ of the field – position that underpins the development of the GENETTA tool.

GENETTA adopts a network approach to encode and represent genetic designs, which has been shown to provide advantages in visualising and analysing circuit features.^4^ A network approach provides comprehensible and structured access to complex data, which often contain relationships that are not immediately apparent. The tool presented here also allows for visualising and modifying genetic designs by providing the user with several views into the underlying data.

## GENETTA

GENETTA is a tool that can be accessed through a web browser and also downloaded for private installation (see Accessibility for details). It aims to help analyse, visualise, manipulate, and modify existing design data. Users will upload their designs using the modify graph tab when they first access the tool. The modify design tab acts as a dashboard for uploading, exporting and removing design data. Designs can be uploaded as a file, pasted, or linked to the tool. Once uploaded, the tool will convert the designs into internal knowledge graphs with which users can interact. A user can upload multiple designs that will only be accessible to that specific user via login.

While GENETTA can handle both GenBank and SBOL^5,6^ files, we strongly recommend using the latter because it captures design information more accurately and robustly in a structured format. SBOL allows for richer data formalization, including functional and structural information. However, even if users are unfamiliar with SBOL, they can still interact with their designs in the tool regardless of the original format. GenBank files can even be uploaded and saved as SBOL after modification. The evolution of data formats operates independently from GENETTA, with its performance improving in tandem with the enhancement of the underlying data format. This way, future endeavours aimed at capturing progressively intricate data, including multicellular designs,^7^ could discover a valuable ally in GENETTA for the analysis of such complex information.

In this version, the three main functionalities of GENETTA are described in the following use cases.

### Use case: dynamic data visualisation

**Figure 1:**
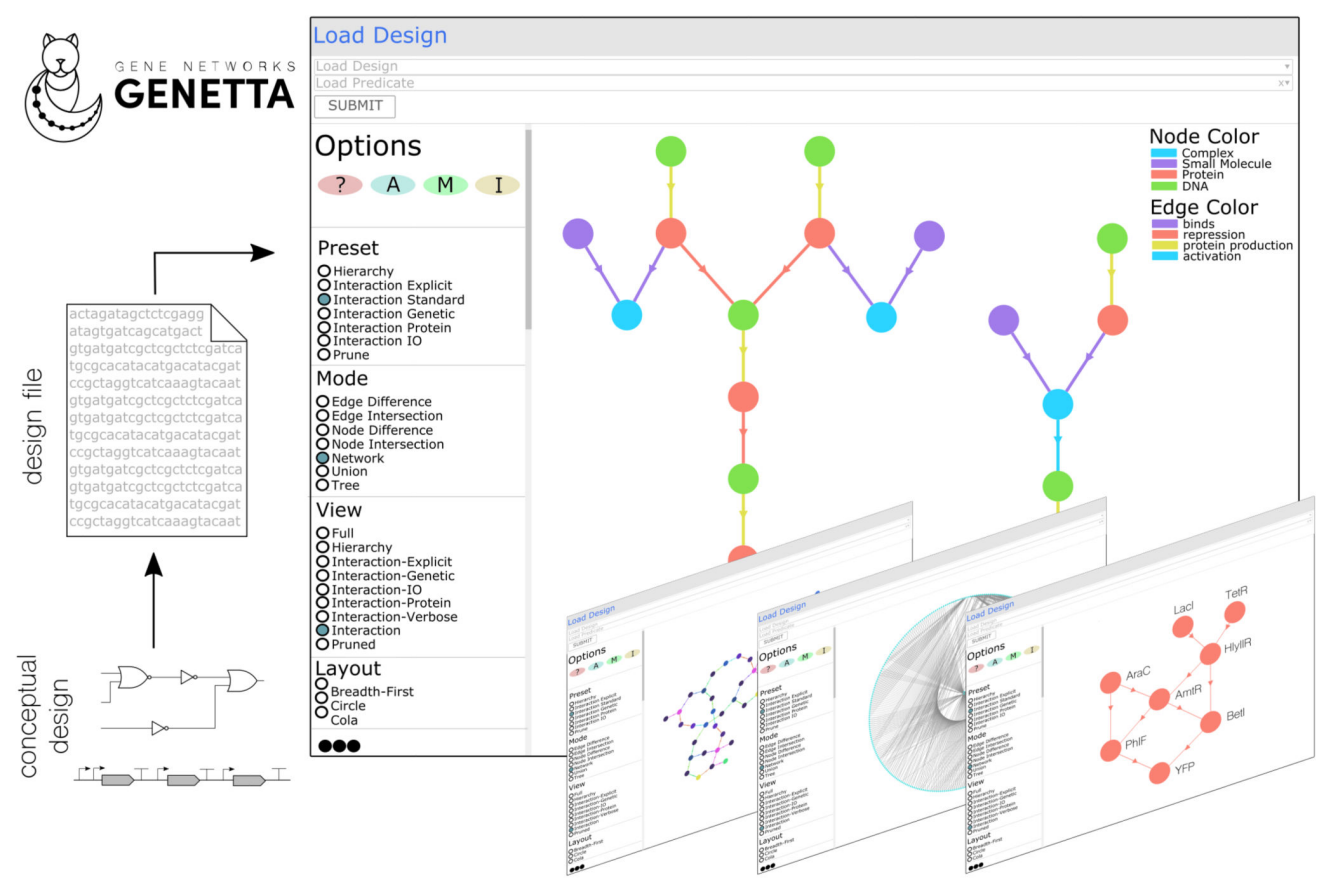
The GENETTA tool. Gene circuit designs are loaded into the tool. The workspace has a column for customisable options to the left and the visualisation canvas to the right. As an example, the design 0xF7 of Nielsen et al.^8^ has been loaded, and it is displayed on canvas using the preset “interaction standard”, the mode “network”, the view “interaction-verbose” (and other options not shown in the screenshot). Options are used to access the documentation (?), further layout customisation (A), manual controls (e.g., colours) and technical information about the network (I) (e.g., number of nodes and connections). The screenshots at the bottom right show other design visualisations.

The visualiser is the standard tool (within the application) to visualise genetic designs as networks. Furthermore, the editor and database tools discussed later extend the features discussed here. The first step is to select an uploaded design from the dropdown menu. Once a design is loaded, the corresponding network will be displayed on the canvas (Figure 1). It is worth noting that the visualisation tool can load multiple designs and integrate them into a single graph. When loading multiple graphs, the load predicate determines how they are connected, with options for *Union* (all data in all graphs are loaded), *Difference* (elements that only exist within one graph are loaded), and *Intersection* (elements that exist within all graphs are loaded).

After loading the graph, it appears in the graph panel, enabling users to access and inspect its information dynamically. While static visualizations like SBOL-Visual^9^ condense a design’s single facet into a diagram, networks serve as data structures that offer comprehensive analysis—not just visualisation. Depending on the chosen view, nodes depict individual data points within a design, like genetic parts, interactions, or metadata. Edges illustrate connections between design data, such as interactions between entities or structural relationships in a hierarchical representation. In essence, networks allow the visualization of complexity. Edges will indicate the shortest path connecting the two linked nodes.

The *Options* panel begins with four buttons that allow users to customise their graph visualisation. *Documentation (?)* provides information on how each graph option affects the projected graph. *Layout customisation (A)* provides further customisation of the layout algorithm used to calculate the positions of nodes on the canvas. *Canvas Changes (M)* allows manual manipulations of the canvas and graph, such as colour changes and node removal. Finally, *Network Information (I)* provides graph-centric technical information from the node and edge count to more specific information, such as the number of components. However, the graph options discussed will provide the main changes to the graph canvas.

*Presets* are collections of preferences (discussed below) that provide a specific graph instance, such as showing hierarchy or protein networks. *Modes* are similar and allow for further data manipulation. For example, *Node Difference* displays the difference between two graphs by removing common nodes, while *Node Intersection* displays their intersection by removing non-common nodes.

*Views* are data projections focusing on a specific aspect by rearranging, aggregating, or making inferences about the complete network, for example, showing the interaction between entities or parts hierarchy. *Layouts* are algorithms to calculate the coordinate positions of nodes on the screen and can be broken into three types: force-directed, hierarchical, or geometric.

Other options not displayed in Figure 1 include colour, text, size, and shape based on features and data types within the network. For example, colours that map to genetic roles. Additionally, users can export the visual and data representations of the current projection. Finally, the legend maps information within non-textual mediums such as colour to values and is automatically generated from changes made within the options panel. All preferences and options are fully described in the tool’s documentation.

### Use case: manually edition of designs

**Figure 2:**
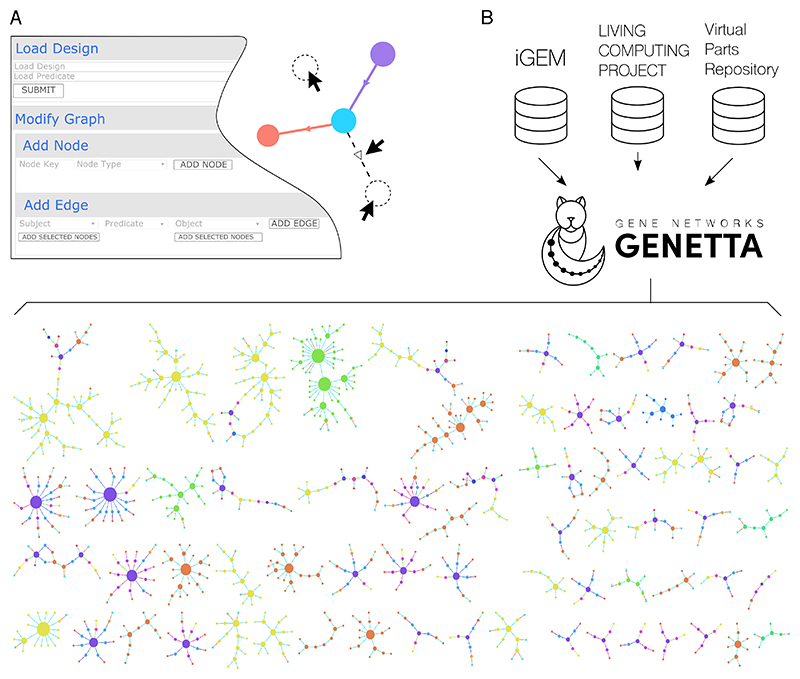
Design editor and data integration using GENETTA. **A**. The editor menu allows users to add nodes and edges to an existing design and to register the type (nodes), subject and predicate (edges). **B**. Example of data integration. GENETTA integrates data from three major synthetic biology repositories: the iGEM parts registry, the Living Computing Project (where CELLO^8^ designs are) and the Virtual Parts Repository. Connections are made between related data, and duplications are removed. Therefore, GENETTA allows for querying networks generated from entire repositories.

GENETTA allows users to manually edit networks while automatically updating the underlying structure (Figure 2A). For instance, if a user adds a new interaction between entities, the editing changes will be automatically reflected in the design data format.

Nodes can be added directly to the network by providing several required and optional values. The first requirement is a unique name for the node (ideally, a URI referencing an existing resource). For example, to add the *lacI* coding sequence from the IGEM parts repository, the user would use the key https://synbiohub.org/public/igem/BBa_R0010/1. The second requirement is the node type, which categorises the information added to the design. The type “ CDS “ would be selected for the *lacI* gene. Metadata such as sequence data can be added as optional properties of the new node. When submitting nodes to be added to the network, GENETTA checks the node key to ensure it resolves to a valid resource (e.g., https://synbiohub.org/public/igem/BBa_R0010/1). If the URI provided does not resolve to an accessible resource, GENETTA will try to find a set of candidates that could be used instead. Matching is achieved using sequence and name matching with databases such as the IGEM parts repository. The editor will then offer the original key with alternatives that can be selected and added to the network.

Users can integrate edges representing relationships between nodes in their network design. The first step is selecting a predicate representing the connection between two data points. The predicate options available depend on the current projection. For instance, if the interaction graph is projected, options such as repression, activation, and genetic production will be provided. The subjects involved in the relfationship are filtered and presented based on their types and whether they are valid with the chosen predicate. For instance, if the user selects “protein production” as the predicate, the input node must be a coding region, and the output subject must be a protein node. The filtering process ensures that invalid information is not inadvertently added to the network via a projection that does not support it, making integration easier. After defining the abstract edge between two data points, the user can submit the data. However, since the projection may not represent the underlying data structure, it may need to be expanded and restructured to ensure compatibility. An algorithm is available for each process of creating a projection graph, so the user does not need to provide additional commands. The data will be automatically integrated into the design, and the projected graph will be updated to reflect the changes made.

### Use case: integration of design databases

Connecting to databases has the advantage of consolidating all available information about a physical or conceptual entity in one location. GENETTA’s internal knowledge graph captures this information and enables analysis across the complete design dataset. For instance, one dataset might not explicitly contain information about interactions between records. In contrast, a second dataset could use many genetic parts encoded within the first and explicitly capture those interactions. Any missing information in a design can be identified and included by linking these datasets.

Figure 2B illustrates how GENETTA integrates three databases - the IGEM parts repository (http://parts.igem.org/Main_Page), the Living Computing Project (https://synbiohub.programmingbiology.org/browse) instance of SynBioHub^10^ (a repository for Cello designs), and the Virtual Parts Repository^11^ (VPR) - and visualises the unified dataset. GENETTA creates a dataset from each source by selecting only the desired records (for example, removing duplicates or records without part descriptions), after which the data is merged into a single network. This process captures two additional pieces of information: synonyms and derivatives. Synonyms are duplicates either between datasets or within datasets. To avoid adding duplicate nodes to the network, a second node is created to indicate that the two records with different names are synonymous. When records are not identical but very similar (particularly in sequence), both records are integrated, and an edge is added to indicate the level of similarity. These similarity edges can be used to identify potential derivative parts.

As a result of this integration, the visualisation consists of multiple disconnected networks where edges represent interactions, synonyms, and derivatives between nodes representing genetic parts. Unlike previous visualisations, the network comprises several components because it does not represent a single design but rather many genetic parts that may have no relationships (or are unknown).

## GENETTA accessibility

GENETTA can be accessed using two methods. Firstly, the most user-friendly way is to use the hosted application: http://biocomputation-cbgp.github.io/genetta. The landing page asks first for setting up a username and password, and then shows the tool along with examples, demos and videos - along with other technical information.

Alternatively, the application can be launched locally using the repository here: https://github.com/Biocomputation-CBGP/genetta-frontend. The repository describes several methods for launching the application depending on requirements are resources. Locally hosting GENETTA may appeal to users using large data structures as some visualisation aspects can be computationally intensive.

Within GENETTA, a more extensive tutorial series describes the application from both a user and technical view. The user view describes practical usage, while the technical discusses how certain features are achieved. Finally, the application also contains several example designs which can be quickly uploaded. These examples range in quality and focus, such as logic gates,^12^ circuits,^13^ part definitions and SEVA vectors.^14^
